# Extended tests for evaluating post-traumatic brain injury deficits in resource-limited settings: methods and pilot study data

**DOI:** 10.3389/fneur.2024.1397625

**Published:** 2024-06-12

**Authors:** Mulugeta Semework, Tsegazeab Laeke, Abenezer Tirsit Aklilu, Abraham Tadele, Yordanos Ashagre, Peter Teklewold, Angelos G. Kolias, Peter Hutchinson, Abel Balcha, Dagnachew Yohannes, Getaw Worku Hassen

**Affiliations:** ^1^Zuckerman Mind, Brain, and Behavior Institute, Columbia University, New York, NY, United States; ^2^Neurosurgery Unit, Black Lion Specialized Hospital, Department of Neurosurgery, College of Health Science Addis Ababa University, Addis Ababa, Ethiopia; ^3^Department of Neurosurgery, AABET Hospital, St Paul’s Hospital Millennium Medical College, Addis Ababa, Ethiopia; ^4^Zewditu Memorial Hospital, Addis Ababa, Ethiopia; ^5^University of Cambridge, Cambridge, United Kingdom; ^6^Wollo University, Dessie, Ethiopia; ^7^Hawassa University Comprehensive Specialized Hospital and College of Medicine, Hawassa, Ethiopia; ^8^Department of Emergency Medicine, Metropolitan Hospital Center, New York Medical College, New York, NY, United States

**Keywords:** traumatic brain injury, TBI assessment, brain injury assessment, TBI tests, LMIC brain injury

## Abstract

**Introduction:**

Traumatic brain injury (TBI) is one of the leading causes of all injury-related deaths and disabilities in the world, especially in low to middle-income countries (LMICs) which also suffer from lower levels of funding for all levels of the health care system for patients suffering from TBI. These patients do not generally get comprehensive diagnostic workup, monitoring, or treatment, and return to work too quickly, often with undiagnosed post-traumatic deficits which in turn can lead to subsequent incidents of physical harm.

**Methods:**

Here, we share methods and results from our research project to establish innovative, simple, and scientifically based practices that dramatically leverage technology and validated testing strategies to identify post-TBI deficits quickly and accurately, to circumvent economic realities on the ground in LMICs. We utilized paper tests such as the Montreal cognitive assessment (MoCA), line-bisection, and Bell’s test. Furthermore, we combined modifications of neuroscience computer tasks to aid in assessing peripheral vision, memory, and analytical accuracies. Data from seventy-one subjects (51 patients and 20 controls, 15 females and 56 males) from 4 hospitals in Ethiopia are presented. The traumatic brain injury group consists of 17 mild, 28 moderate, and 8 severe patients (based on the initial Glasgow Comma Score). Controls are age and education-matched subjects (no known history of TBI, brain lesions, or spatial neglect symptoms).

**Results:**

We found these neurophysiological methods can: 1) be implemented in LMICs and 2) test impairments caused by TBI, which generally affect brain processing speed, memory, and both executive and cognitive controls.

**Discussion:**

The main findings indicate that these examinations can identify several deficits, especially the MoCA test. These tests show great promise to assist in the evaluation of TBI patients and support the establishment of dedicated rehabilitation centers. Our next steps will be expansion of the cohort size and application of the tests to other settings.

## Introduction

Traumatic brain injury (TBI) is a type of acquired brain injury due to sudden trauma from objects hitting the head with or without actual skull fracture. It can cause brain tissue damage and functional deficits (1).

The Centers for Disease Control and Prevention (CDC) broadly defines TBI as “an injury that affects how the brain works” (2). The widely accepted approaches use clinical severity to classify TBI as mild, moderate, or severe. Some maintain these severity definitions are arbitrary (3). Accordingly, some classify head injuries, not by their clinical severity, but rather based upon (1) structural damage; (2) mechanism of injury, and even (3) prognosis (4).

TBI is the leading cause of all injury-related deaths and disabilities within low to middle-income countries (LMICs), and according to the 2017 publication from the Lancet Neurology Commission TBI will remain within the top three causes of injury-related death and disability until 2030 (6).

LMICs account for 85% of the world’s population. Each year, 50 million TBIs occur in LMICs, compared to 18 million in high-income countries (7). Within LMICs, TBI is a significant cause of hospitalization, death, and disability (8, 9), and the mortality rate from TBIs is 3–4 times higher than the reported rate in high-income countries (10). Additionally, LMICs have greater odds of disability and twice as much death from severe TBI (5, 11).

These disparities are discussed here per the standard classification of the severity of TBI, the Glasgow Coma Scale (GCS), which defines GCS of 13–15 as mild, 9–12 as moderate, and 3–8 as severe (12). The situations on the ground (discussed later in the limitations section), such as lack of trained personnel, subject unavailability for long-term follow-up etc., did not allow outcome measures using GCS, Glasgow Outcome Scale (GOS) or the Glasgow Outcome Scale Extended (GOS-E) tests.

GCS is universally used to on acute medical and trauma patients as a tool for objectively describing the extent of impaired consciousness. Using three levels of responsiveness, eye-opening, motor, and verbal responses, the test aggregates its results providing an overall severity and has been incorporated in numerous clinical guidelines and scoring systems for victims of trauma or critical illness. Clinically, GCS is used to help early management such as securing the airway, patient transfer, neuroimaging, discharge, and monitoring clinical course (17). Studies have shown that increased mortality is associated with decreased GCS score (13), with each assessment yielding more information than the aggregate total score (14). This is distinction is further demonstrated by studies which have shown that level of consciousness is not accurately reflected by total GCS scores, suggesting the need to consider individual subscale behaviors and more comprehensive assessments when evaluating TBI severity (15, 16).

Given the fact that GCS scores can be influenced by several factors such as sensory/language barriers, current treatment or other injury effects, this powerful method or other clinical prognostic tools should not be used solely to predict individual patient outcomes (17).

Moreover, the GCS classification system does not account for visual deficits (18), cognitive deficits (19, 20), and cognitive aging (21), all of which can be present either initially or in the immediate post-traumatic period.

These issues can be harsh especially in LMICs where even such classifications cannot made reliably due to factors such as lack of trained professionals, the patient not arriving at the hospital immediately after injury or not being followed properly.

Furthermore, the lack of resources, research efforts, and funding for health care contribute to the disparities of LMIC’s compared to high-income countries (9). Although TBI causes half of trauma deaths (22–25), LMICs lack data on head injury in general (26), the morbidity, and mortality, of head injury, causing underestimation of the problems (27).

Moreover, the epidemiology and management strategies of TBI in LMICs only by few studies and TBI patients in LMICs suffer from lack of access to imaging, patient monitoring equipment, surgeons, and rehabilitation services (7).

At the same time, economic responsibilities for self and family members force most patients with mild to moderate TBI to return to work quickly with what are almost certainly undiagnosed post-traumatic cognitive deficits. This could lead to subsequent accidents in their regular jobs of operating cars, taxis, trucks, and other heavy machinery, making consequential decisions, and causing a vicious circle of potentially significant injuries. These chances are high as LMICs have risk factors for TBI causes (such as motor vehicle crashes) which occur at a higher prevalence and getting medical care that address associated health effects does not occur at the same rate as HICs (28).

Given the importance and difficulty of assessing cognitive deficits of TBI patients, we chose Ethiopia as a demonstration site for developing a method for cognitive screening in TBI patients in an LMIC (19, 20). Ethiopia is the second most populous nation in Africa, located in the sub-Saharan region, where the estimated TBI incidence is 801 per 100,000. The mortality rate for severe TBI (severity as determined by the aforementioned GCS scale) is approximately 50% (29, 30). TBI is particularly frequent in younger people (31). Significantly, the country has more than 70 million citizens younger than 30 years of age (32, 33). This fact has contributed to a substantial toll on society ranging due to TBI-related disabilities and deaths (34, 35). Given that Ethiopia’s population is predicted to double in the next 30 years, reaching 210 million by 2060 the overall toll from minimal TBI diagnostic workup, monitoring, and rehabilitation and in some cases even under diagnosis of TBI should be expected to be a substantial heath crisis (36). Studies like this one will be valuable as more LMICs follow the same trend, especially in Africa, where population growth is expected to increase in the next 40–50 years (37).

A recent study in Ethiopia of 4206 deaths brought to the Forensic Pathology Department. Menelik II Hospital has shown that accidents, homicide, and suicide caused 67% of them (38). Of these, accidental death contributed to 40%, of which traffic-related cases were close to 70%. Another postmortem study of almost 10,000 patients, all who died within 72 h of emergency department (ED) presentation revealed that head injury is the most common cause of mortality (21.5%) of all deaths in Ethiopia (39). In the country, the majority of post-traumatic hospital visits are mainly from head injury (34). The two primary sources of TBI are violence and road traffic accidents (RTA) (40). One of the major hospitals receiving these patients involved, Black Lion Specialized Hospital (which is also one of the very few tertiary hospitals giving neurosurgical services in the country), recorded that the commonest cause of death in its ED is head injury (39). A study in Nigeria (the most populous country in Africa) corroborates the same story (41).

Here, we examined the utility of a number of different tests to determine the severity of the cognitive deficits in patients with TBI. We found that the MoCA, which can be administered by easily trained, non-medically educated personnel, was the most effective method for assessing cognitive deficits in TBI patients.

## Methods

### Database

We studied 51 TBI patients who had follow-up visits in outpatient neurosurgery clinics and 20 controls, all of whom signed a consent form. The cohort had 56 males and 15 females. Age ranged from 14 to 65, with 18 of them being young people (10–24 years of age) and 53 adults [per the World Health Organization (WHO) definition (42)]. The TBI group comprised 17 mild, 28 moderate, and 6 severe patients (based on initial GCS). Several GCS measurements are routinely taken at these hospitals – such as after brain surgery, after days of recovery etc., however all of the TBI classifications in this research used the initial GCS which is administered at admission, post-resuscitation when needed.

The injuries included 22 to either the right or left hemisphere, and 7 bilaterally. The causes of damage were: violence (25 patients), road traffic accidents RTA (19 patients), and falls (7 patients). Controls (20 subjects) are age and education-matched individuals (no known TBI, brain lesions, or spatial neglect symptoms). A table with the full demographic data and paper test results is provided as a Supplementary Appendix A.

We conducted the study in five locations chosen for several reasons, the main ones being geographical coverage and the existence of approved protocols and collaborations. These corresponding hospitals are located in central (Black Lion and AABET, 12 and 46 subjects, respectively), northwestern (Tibebe Ghion, 9 subjects), northeastern (Dessie, 2 subjects), and southern (Hawassa University Teaching and Referral hospital, 2 subjects) parts of Ethiopia.

We performed most of the studies in two hospitals (Black Lion and AABET, 58/71 subjects) in Addis Ababa, the capital of Ethiopia. Black Lion (BL) Hospital is the biggest referral public hospital in Ethiopia (43). The country’s primary health care establishments use referral systems to provide access to community-based health centers before higher hospitals and care options are considered (44).

AABET Hospital is one of the few tertiary hospitals in the country that provides emergency and critical care, orthopedic, neurosurgery, general, and plastic surgery services (45) and it is the only major trauma center in Addis Ababa (46).

Tibebe Ghion Comprehensive Specialized Hospital (TGCSH) is a regional center located in Bahir Dar (the capital city of Amhara National Regional State) in the northwestern part of the country, 565 km from Addis Ababa. The hospital provides different medical services to more than 5 million people in the region (13, 47).

Dessie Referral Hospital (DRH) is one of the largest and frontline public hospitals in Ethiopia’s northeastern part (in Dessie, the administrative town of South Wollo zone), 400 km from Addis Ababa (48). It is a referral hospital serving close to 5 million people (49).

Hawassa University Referral Hospital (HURH) is a tertiary-level hospital located in Hawassa, the capital city of southern Ethiopia (275 km to the south of Addis Ababa). It serves around 12 million people in the Southern Nations, Nationalities, and People Region (SNNPR) and the neighboring Oromia region (50).

The breakdown of subject categories per hospital are as follows: AABET 12 controls and 34 tests, BL 8 tests and 4 controls, TGCSH 5 tests and 3 controls, both DRH 2 tests each.

### Testing environment and data collected

We administered all tests in routine patient evaluation rooms in the corresponding clinics. At least one family member was available to verify demographic and other information before the start of the experiments. After the verification procedure the subjects were not allowed to interact with their families or other participants until after the testing session was completed.

Given the possibility that most subjects have yet to gain experience with paper tests or computer knowledge, we described the tasks in depth. The tests started when the examiner was sure the subjects were able to perform both the paper tests and computer operations without impediment.

All subjects were briefed on experiment goals and procedures and signed a consent form as determined by the specifics of the IRBs. All participants verbally agreed to be photographed and videotaped for educational purposes.

Each session lasted approximately 30–45 min with 3–4 breaks lasting an average of 5 min. Subjects could request a break or terminate the experiment at any time. We did not perform any invasive procedures or administer any medication.

**Figure 1:**
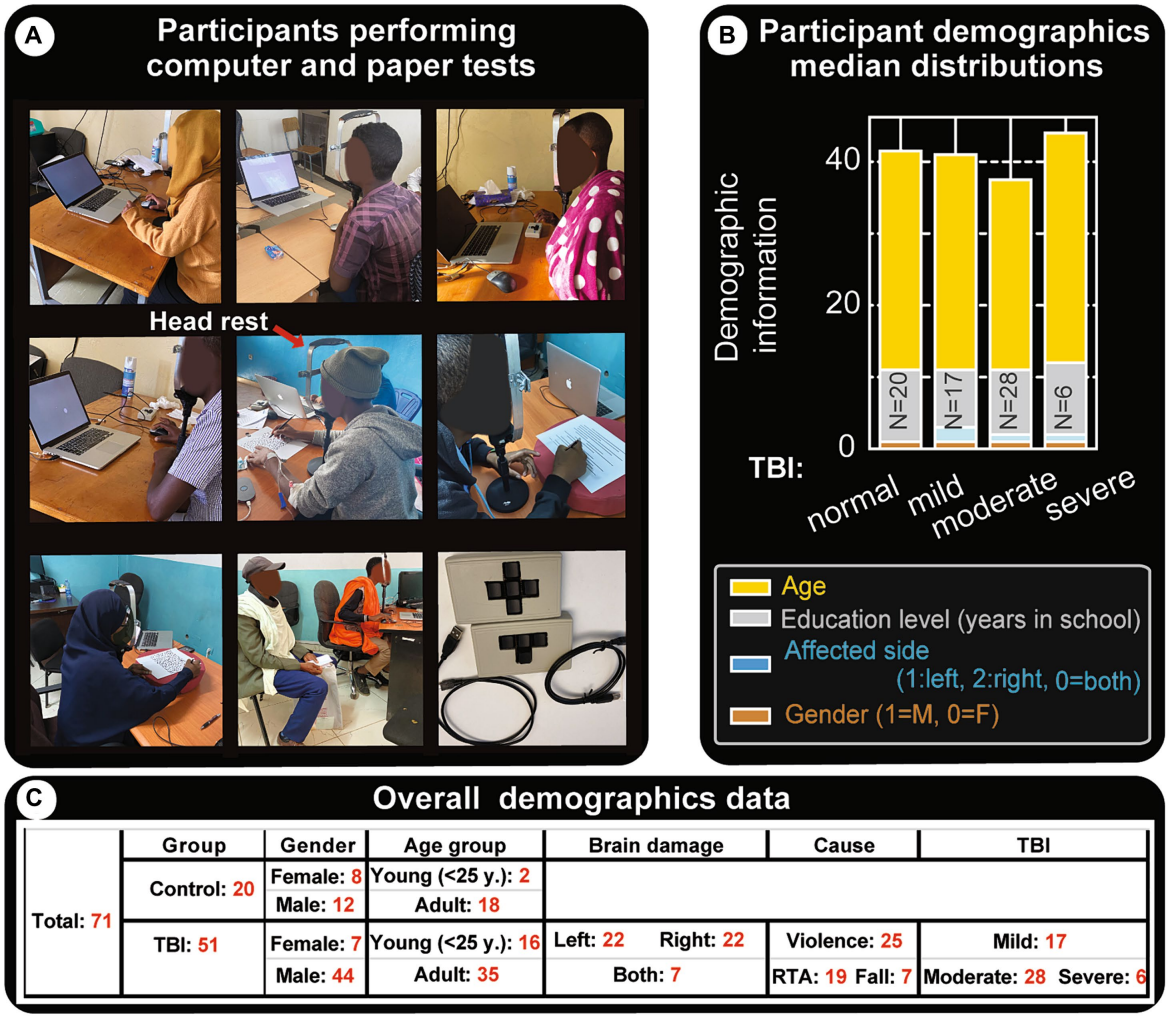
Demographics and experimental settings. **(A)** Snapshots of subjects as they perform paper and computer tests. The experiments are run in regular hospital office settings with at least one family member present to assist with initial interactions, without helping the subject with test performance. The subjects are instructed on what to do and data collection is started once they are confident. In general, the tests require visual focus in the center of their visual field (the center of the screen in computer tests) and have to keep their head from swaying. However, most subjects had difficulties following instructions to not move their heads during the sessions, therefore a chin/head restraint was used (indicated by the red arrow in the middle panel). Bottom right panel shows custom-made 3 and 4 key USB mini computer keyboards that were used for capturing their responses. **(B)** Clinical data and demographics of patients with TBI. This panel shows the type of TBI and median distributions for age, education level, affected brain side and gender. Effort has been made to get more or less comparable representations for these parameters and for each TBI group. Similar heights in the category bar charts indicate the cohorts are fairly balanced. **(C)** Clinical data and demographics: tabular representation of major attributes of participants.

Figure 1A shows research subjects performing paper and computer tasks. Head/chin restraints were used to keep subjects from moving their heads during paper and computer tests (red arrow in Figure 1A, middle panel). This apparatus kept the eyes at ~50 cm from the paper or computer screen. A computer mouse and a novel four-key mini keyboard input instrument specifically made for this study (Figure 1A, last panel) were used by subjects to respond to questions without repeated searching or looking down at the keyboard after initial instruction. For computer tests, each subject was provided with initial training and assessment before administering the actual test. If the subject had any difficulty understanding or performing the task, he/she did not move onto the actual task. For some subjects this meant the computer tests went a little beyond the average 30 min per session and ended up being close to 45 min.

In addition to test performance, clinical data such as time since injury, cause of TBI, and other contributing factors such as alcohol use were also collected.

### Tests

The following conventional/novel clinical vision, memory, and spatial neglect tests were used (ranked in order of ease and overall order of administration).

#### Paper tests

We used and scored three internationally validated paper tests.

**Figure 2:**
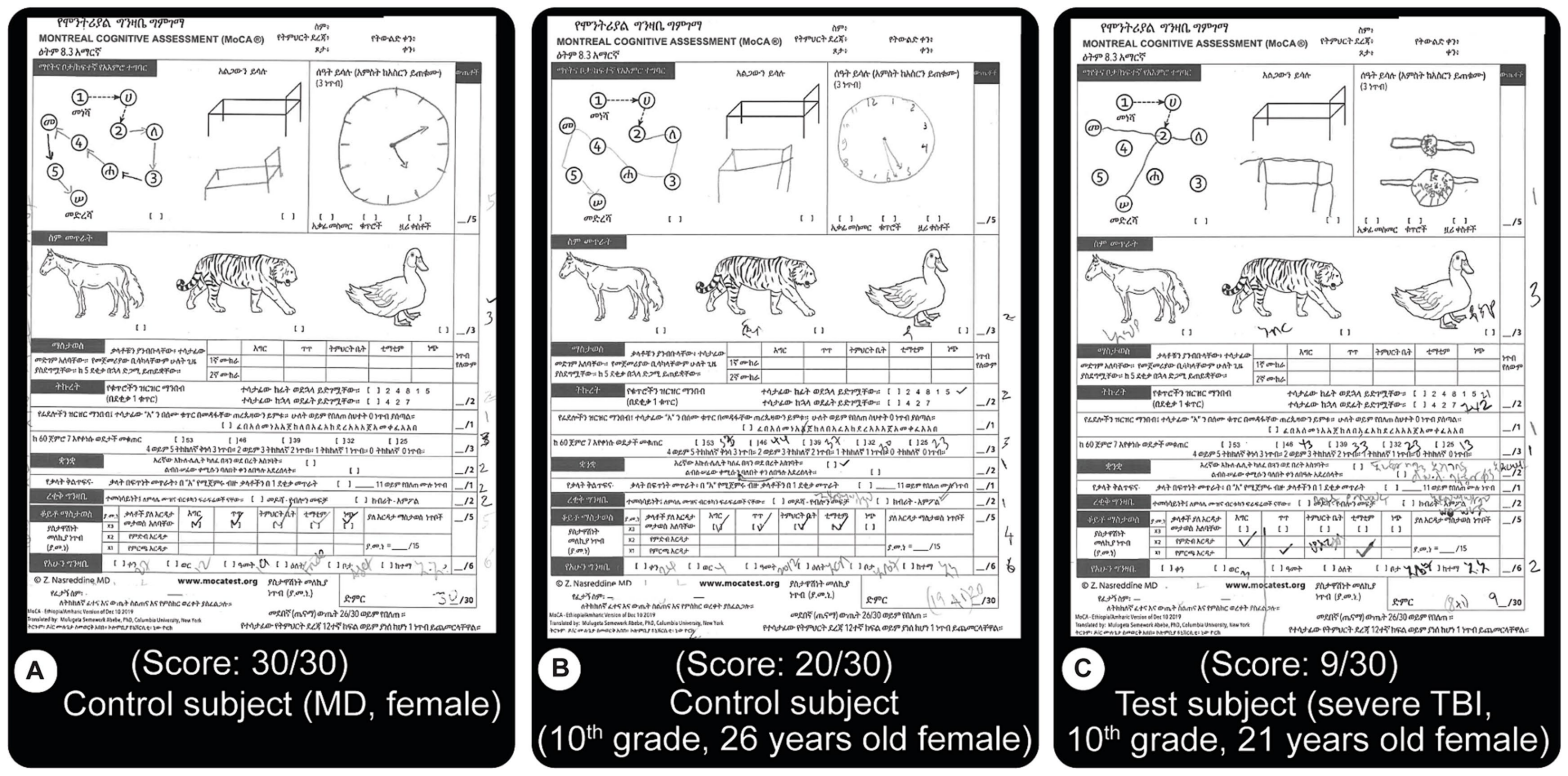
MoCA test results. This test is an internationally validated tool that focuses on motor, memory, language, concentration and orientation performances. Subjects are asked to perform different tasks such as to draw lines from a letter to a number, both increasing, draw objects from memory, name displayed animals, recall specific words, etc. The version used here is an official translation of the test to the national language of Ethiopia, Amharic. A score of 26 or more out of the possible 30 is considered normal. **(A)** Normal score from a control subject (MD level education). **(B)** Close to good score (20/30) result from a control subject **(C)** Test result from a patient with severe TBI showing a lower score (9/30). Both **(B,C)** have 10th grade education. Most of the errors from TBI patients were related to memory issues.

Montreal cognitive assessment: this internationally-validated assessment evaluates cognitive function and it includes tests of orientation, attention, memory, language, and visual–spatial skills. For this project, it has been officially and professionally translated into the national language of Ethiopia (Amharic) (currently available at MoCA’s official website: https://www.MoCAtest.org) (see Figure 2). The translation included necessary changes for cultural appropriateness. We used this new version for all subjects and consistently administered it as the first test.

Because spatial neglect commonly causes problems in daily routines such as crossing the street, we decided to assess spatial neglect. The other two paper tests used are the most common clinical tools to assess spatial neglect, both probing the same underlying cortical deficits (51).

**Figure 3:**
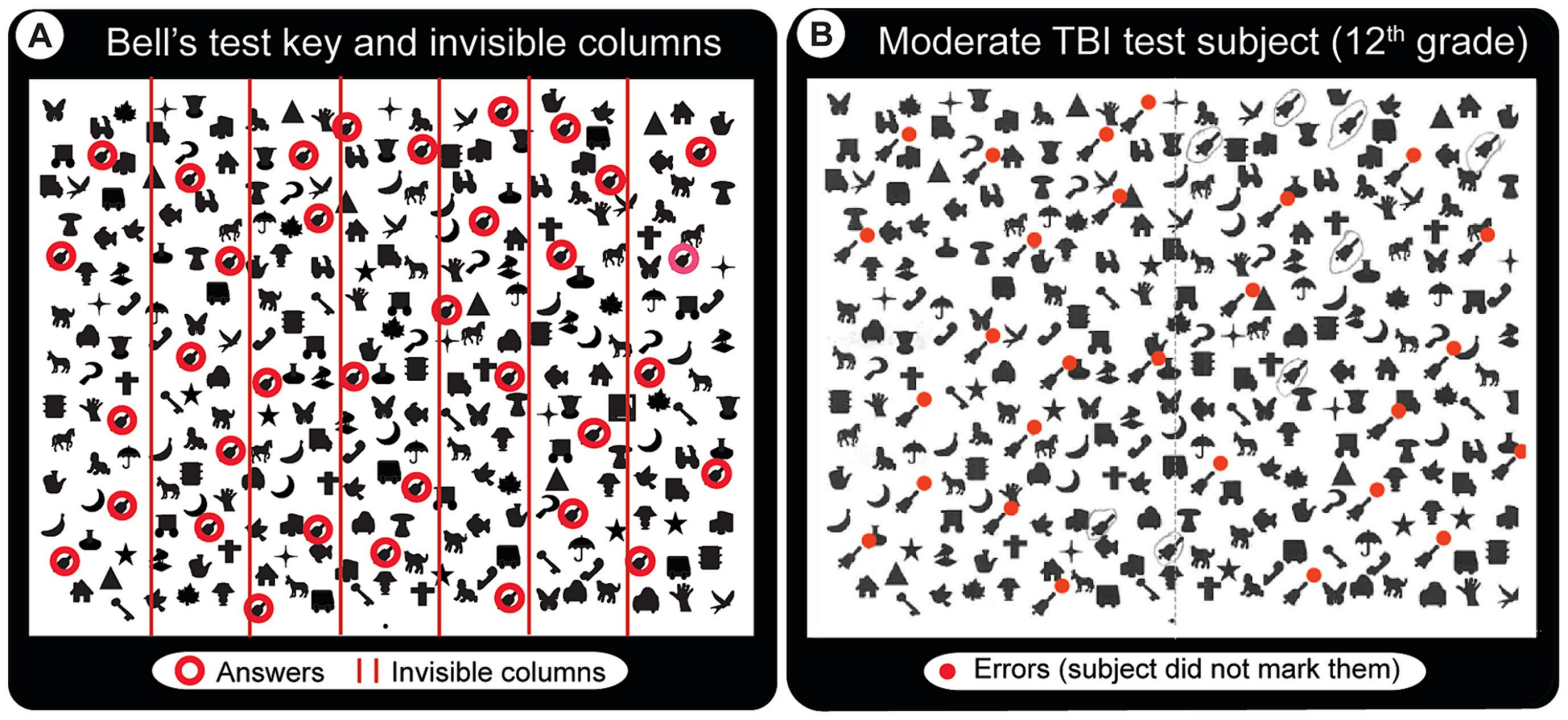
Bell’s test solution key and a sample result from a patient with moderate TBI. The task requires locating and drawing a line around each of the bell-shaped images in an array of other images without moving the head. **(A)** Solution key. Red circles (not visible in the actual test paper) indicate the 35 bells that the subject has to find and circle. They are laid randomly in 7 invisible columns (5 per column, red lines indicate the column boundaries). **(B)** Abnormal Bell’s test result from a patient with moderate TBI who appears to have spatial neglect mainly for the lower left visual field (red dots indicate missed bells).

**Figure 4:**
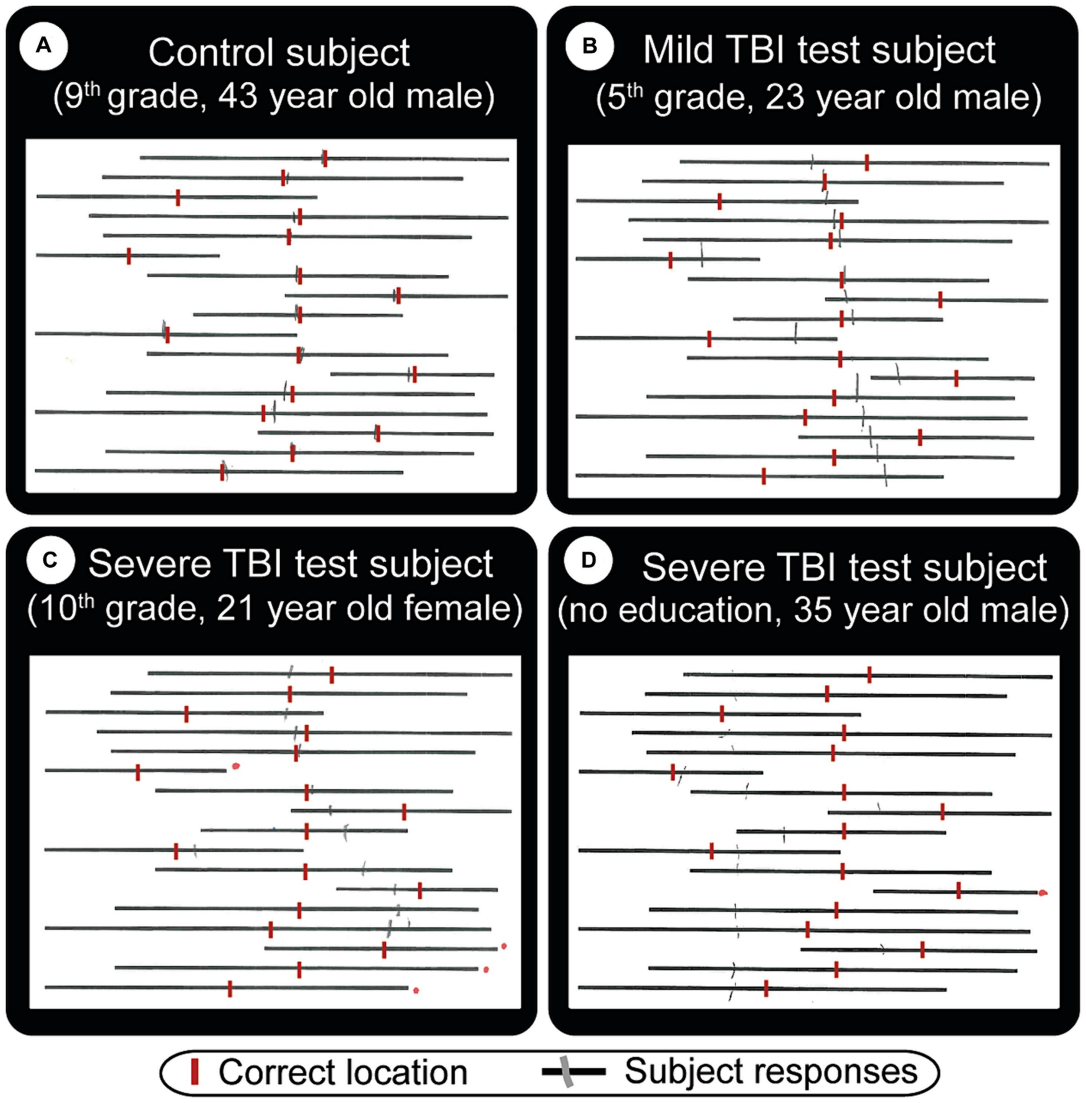
Straight line bisection test result examples. The subject’s task is to draw a vertical tick mark where he/she thinks the middle point of each line is. These are indicated by red vertical lines (manually added here for visual comparison). Gray (pencil) lines are actual subject responses. Depending on what kind of visual problems the subject has, the middle point can be difficult to estimate. **(A)** Normal subject who shows minor errors. TBI patients (**B**, mild) and (**C**, severe) show strong cases of left neglect, in that they both made lines that are mostly to the right of the ideal location. Subject **(C)** did not cross 4 lines (red dots to the right of missed lines). **(D)** Severe TBI patient with right neglect, who also missed one line, has a very big total error (errors are easier to see in Figure 8B).

1. Bell’s Target Cancelation task (52) (spatial neglect task 1): this is a visual search and mark task on an A4 size sheet of paper, which has 40 distractors & five targets (bells) in each of seven invisible columns. Patients circle all bells, and a score is created from the difference in omissions on ipsi-minus contra-lateral sides (Figure 3). Three or more ipsilateral vs. contralateral omissions are taken as having spatial neglect (23). To accomplish this (and make the test usable by the local population), we created a new version of the Bell’s test by making an entirely new library of images to Figure 3.
2. Line-bisection task (53) (spatial neglect task 2): the patient makes a small pencil mark in the middle point of each of the multiple lines with varying lengths on A4 size paper. Left visual neglect will cause subjects to make right-ward errors, and vice versa for right neglect. In the classic line-bisection task (53), the patient makes a small pencil mark in the middle of each of the 20 lines with varying lengths (100, 120, 140, 150160, 180, and 200 mm). Deviation percentages over all lines (both left and right deviations) are scored. Having an ipsilesional deviation above 9.5 percent is defined as having spatial neglect. This number is selected because it had a confidence interval above 99% for the control group (42). In our initial test, data from the first 10 subjects made it clear that short (18 mm) slanted lines were inadequate or very ineffective in evaluating deviations. With the extended horizontal line version utilized, we found discernible differences within and between patient and control subject groups (Figure 4). For simplicity and clarity, the data presented here uses the simple metrics of summing up of all negative and positive errors (millimeters away from an ideal middle). After we gather statistically valid control data, the deviation percentage method will be utilized.

#### Computer based tests

**Figure 5:**
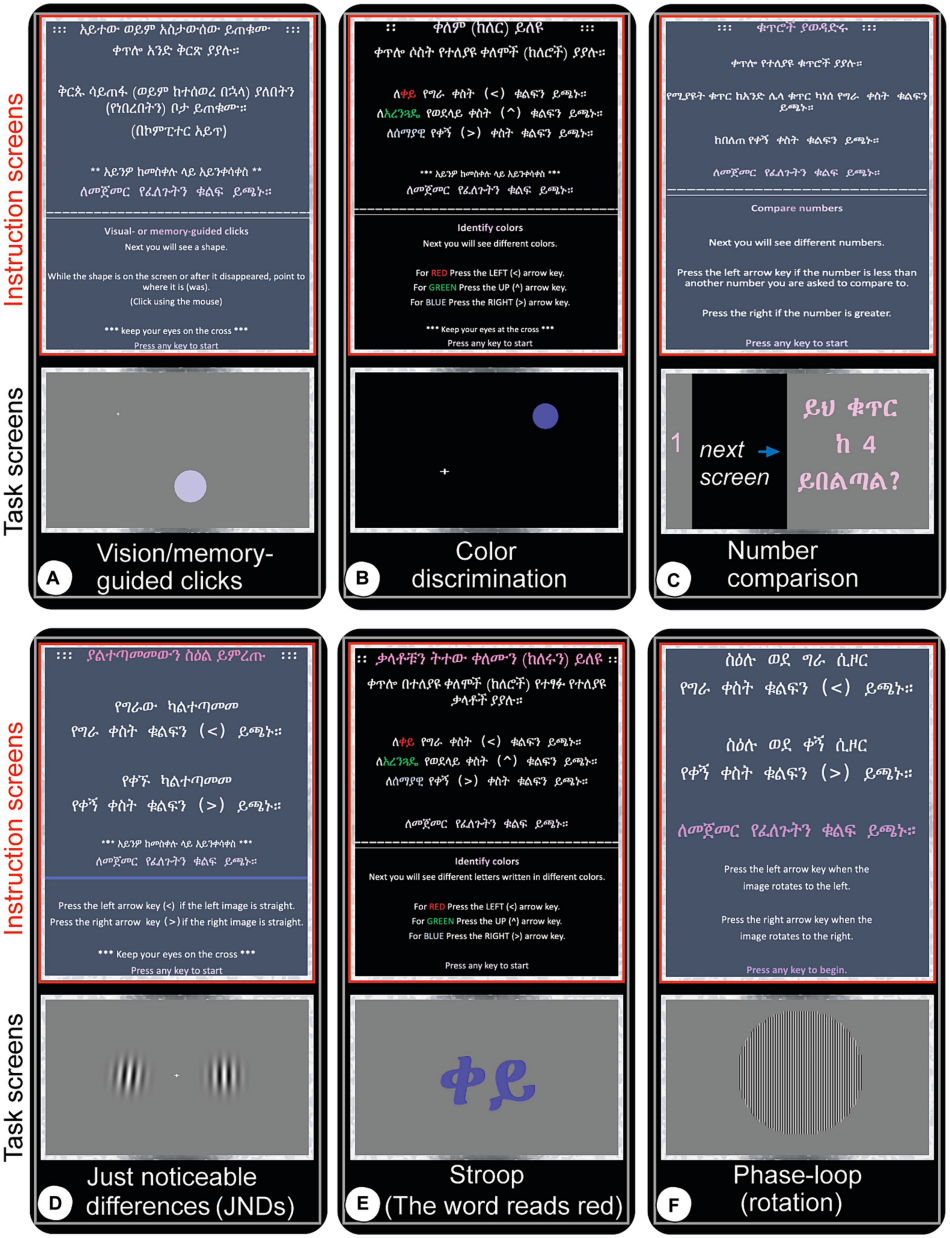
Screen captures (real-time snapshots) of computer test instructions and actual experiments as they are being administered. Once the participant expresses confidence in taking the test, the experiment is started with the first display being the written instructions (red-boxed, top panels in each test panel) followed by the actual experiment (gray-boxed, bottom panels). The subjects wait for a central target to appear and are instructed to keep their eyes focused there while using peripheral vision to perform the tasks and respond with a mouse movement and click or custom keyboard click. Experiments are self-terminated when all trials are completed. **(A)** Vision and memory-guided clicks: move the mouse to a target location where a circular stimulus appears randomly while it is still displayed (visual), or once it appears quickly and disappears (memory). **(B)** Color discrimination: red, green and blue circles appear left, above or right of fixation or at random location and order and the subject has to click the left, up or right arrow keys for red, green and blue colors, respectively. All subjects are tested for color-blindness to these colors and none of the subjects had difficulties separately identifying them. **(C)** Number comparison: click left arrow key if the number you just saw is smaller than the number that was quickly displayed a moment ago, or the right arrow key if it is greater. **(D)** Just-noticeable differences (JNDs): two circular images filled with nearly/vertical bars (Gabor patches) are displayed left and right of the central fixation point. Click the left or right arrow key if the left or right image is vertical, respectively. **(E)** Stroop test: this task measures the level of accuracy in dissociating color from information a given text displays. For instance, in this snapshot, the Ethiopian (Amharic) word displayed stands for ‘red’ but it is written in blue. The subject has to press the right arrow key which represents the color blue, not the left one which represents red color. **(F)** Phase-loop: press the left arrow key if the image seems to rotate left, or right arrow key if it is perceived to move to the right.

We wrote 6 computer based tests specifically for this study in Python which we tested on 10 volunteers before using them on study subjects (Figure 5) – prior to any real data recording.

At the start of each of the 6 computer tests, the experimenter described the task verbally and demonstrated a few trials. Once the subject is confident, a graphic display describes the task and user interaction guidelines once more and all efforts are made to maximize performance. For example, in the Just Noticeable Difference (JND) task (Figure 5D), a user must press the left or right arrow keys to select which of the images (Gabor patches) presented, on either side of a central fixation cross is not slanted. Our custom keyboards allowed for the subjects to signal a rapid response.

1. Visual and Memory Performance (Figure 5A): measured with both vision and memory-guided hand movements. Subjects are asked to visually fixate in a middle location in a computer screen (center of visual field) until a circular target appears in random locations on the computer screen. For visual targets, the subject moves a mouse cursor to the peripheral target that has just appeared and clicks inside the visible circle. The memory task requires the subject to make the same response to a briefly presented target. The subjects performance is measured by click location accuracy.
2. Color Discrimination: Color experiments tested the capacity to discern red, green, and blue colors (RGB) using the left, top, and right arrow keys, respectively. There were three versions: (1) Color center (RGB): colored circles appearing at the center of the screen; (2) Color Left–right (LR): colored circles appearing left or right of a central fixation cross; (3) Color Left–right-UP (LRU): colored circles appearing left, above or right of a central fixation cross; (4) Color random (RGB): colored circles appearing in random locations in the computer screen.
3. Just Noticeable Differences (JND) (slanted Gabor patches), choosing the image which is perfectly vertical compared to another image just on the other side of a fixation cross. The fixation cross is located on the center of the screen (Figure 5D).
4. Color and Word Dissociation (Stroop tests) choose and respond to the color of a word you see on the screen, ignoring the word. For instance, the subject press the left arrow key for red color even if the work reads “blue” (written in Amharic letters) (Figure 5E).
5. Motion and Contrast Perception (object rotation, phase loop): press the left or right arrow key depending on which way the subject thinks the central image is rotating. This test also includes various illumination levels (to test contrast sensitivity) and rotation speeds (Figure 5F).
6. Math operations: simple numbers comparison: the subject is instructed to press the left arrow key if the number seen earlier is smaller than the one number most recently seen; Otherwise, press the right arrow key (Figure 5C).

## Results

For relatively fair comparisons, similar participants were included to the different groups (shown in Figure 1B). This study includes 71 subjects (51 patients and 20 controls) (see table in Figure 1C). The median distributions of the most important metrics, age, education level, and affected brain side, are comparable. However, gender is mostly male-dominated (56 versus 15), consistent with world data [some studies conclude the chances of sustaining TBI are 2.22 in males than females (42, 54)].

The MoCA was the most accurate and specific test in evaluating TBI-induced deficiencies, especially memory and general executive thinking deficits. The test corrects for education, i.e., by adding a point to the total score if the subject has 12 years of education or fewer.

**Figure 6:**
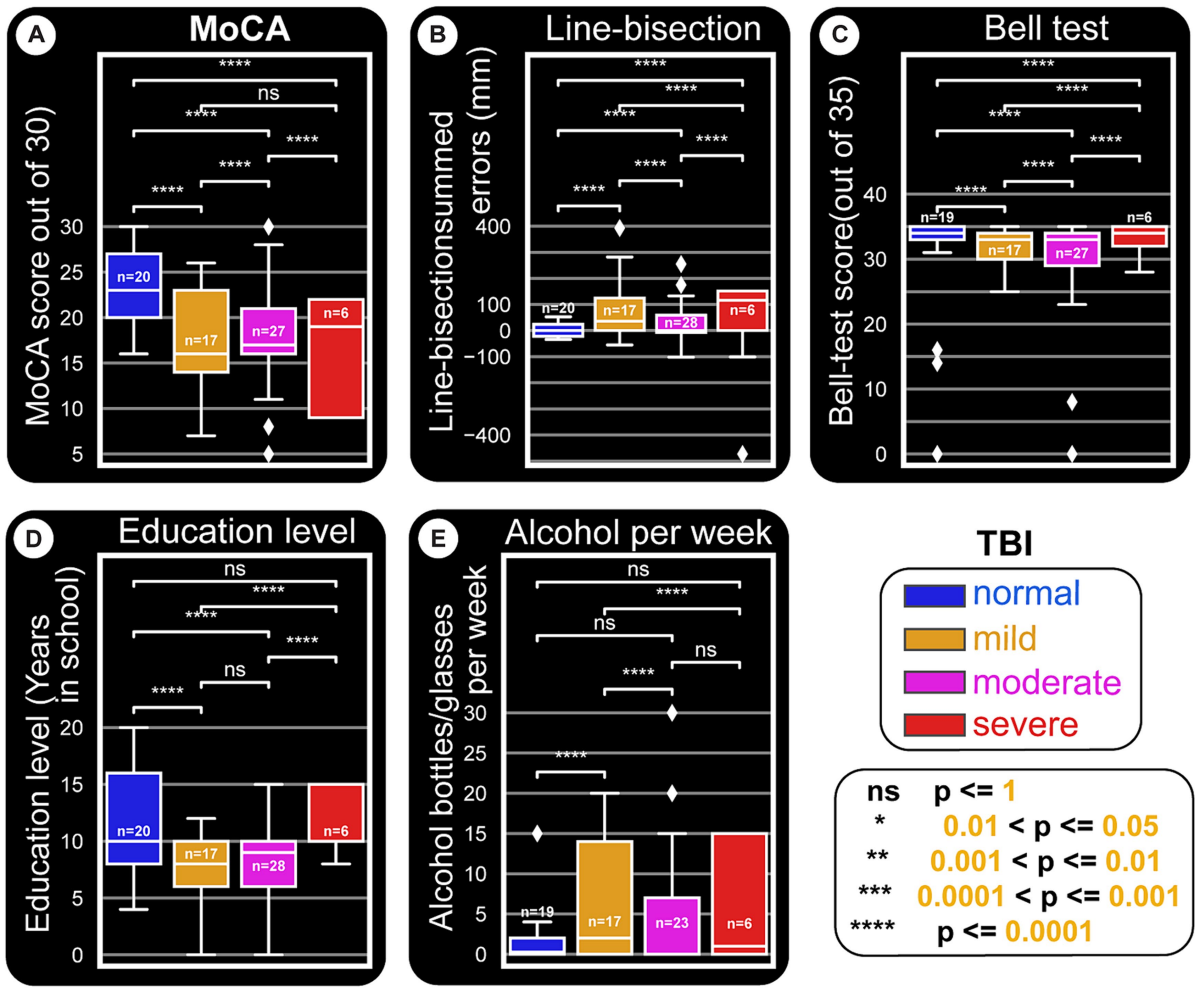
Paper tests and education level and their relationships with TBI types. This distribution figure uses box plots to compare quantitative data from different variables. Straight line segments above each pair of boxes connect significantly different as shown by *p*-values from Mann–Whitney-Wilcoxon two-sided test. The level of these differences are depicted by asterisks (‘*’) or ‘ns’ (not significant). As in other figures, all *x*-axes are set to the main categorization used: TBI kind (normal, mild, moderate and severe). The three quartile values of each distribution are shown by the boxes and the rest of the distribution is presented by the whiskers. These whiskers extend to observations that are within 1.5 interquartile-ranges of the lower and the upper quartile. The mid-point of the data (middle quartile, also called the median) is indicated the middle line in the boxplot that divides the two parts of the box. Outliers are shown by diamond points. **(A)** MoCA score is reduced with severity of TBI damages. **(B)** Line-bisection test scores. The boxplots are plotted against a *y*-axis which depicts the cumulative errors for all line crossings that are left (negative) or right (positive) of the ideal middle for each line. Accordingly, more positively skewed crossings indicate possible left neglect. It appears that patient groups tend to be right or left skewed, owing to the majority of the side of the brain damaged for the cohort. **(C)** Bell’s test score versus TBI damages does not seem to show major differences. However, as in Figures 8B,C, normal subjects seem to have a stable error distribution, while patients (especially moderate and severe TBI ones) show wider and skewed boxplots. **(D)** Education level also seem to be related to TBI damage, in that normal subjects tend to have higher education. Whether this points to a major predisposition to get into TBI situations remains to be seen. **(E)** The amount of alcohol consumed per week appears to be positively related to having TBI in general.

**Figure 7:**
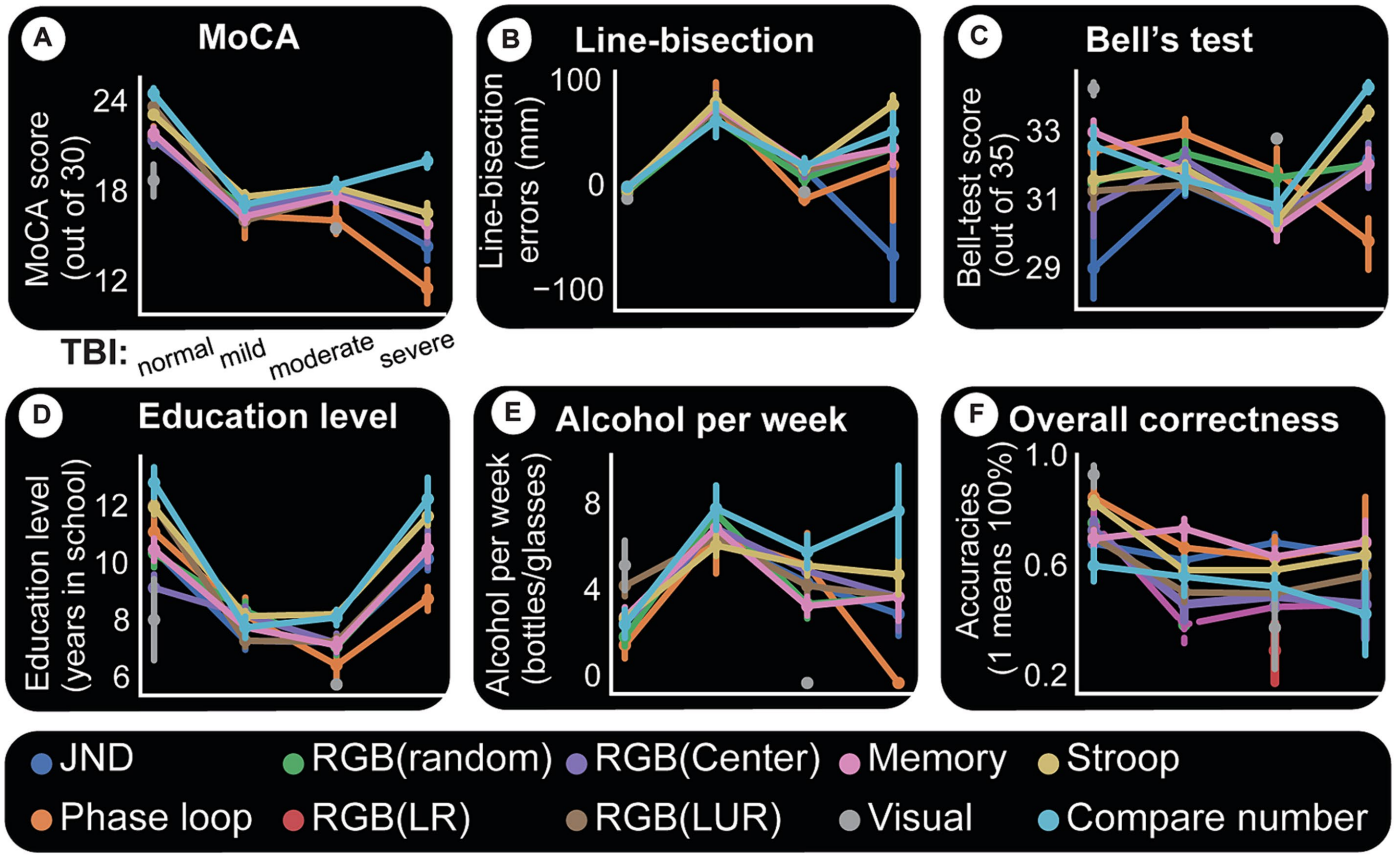
Computer tests and their relationships with paper tests and accuracies. For all panels, *x*-axis is TBI category. Each colored line indicates a specific computer test. Each point in a given line is the mean paper test performance response for the given TBI group (*x*-axis) and for the subjects who took that particular computer test. The uncertainty of the estimate is shown by whiskers (indicating standard error). A simple way to understand this figure is to look at panel **(A)** and see that the central tendency for MoCA scores and all computer tests is to decline with TBI severity. This same panel shows that there is more certainty about this assumption because the standard errors are small. **(A)** MoCA test appears to affect all computer test results relatively the same way. **(B)** Line-bisection performance also seems to follow TBI trends except perhaps number comparisons which show a slight difference for mild patients. **(C)** Bell’s test. One possibly important observation here is a clear spread for normal subjects in performances for different computer tests, indicating better task discriminations. **(D)** Computer tests and accuracy (correctness). *Y*-axis indicates the average accuracy (scaled 0 to 1) for the given cohort. A score of “1” indicates all members of a given group (say normal subjects doing Stroop task) had a perfect 100% accuracy. Overall, TBI appears to cause decline in accuracy and more variability in performance (as shown by whiskers for each category and computer test). Normal subjects perform better in all computer tests, followed by mild TBI patients, etc. **(E)** Alcohol consumption data points to the presence of discernability for computer tests and TBI categories. It is interesting to note here that the two time-consuming tasks, Stroop test and number comparisons, seem to be more discriminatory of TBI categories using alcohol consumption. **(F)** Computer tests and accuracy (correctness). *Y*-axis indicates the average accuracy (scaled 0 to 1) for the given cohort. A score of “1” indicates all members of a given group (say normal subjects doing Stroop task) had a perfect 100% accuracy. Overall, TBI appears to cause decline in accuracy and more variability in performance (as shown by whiskers for each category and computer test).

We found TBI severity correlated with reduced test performance. Figure 2 depicts these observations. A control subject with high education (medical doctor) received a perfect score (30/30, Figure 2A, above 26 is considered ‘normal.’ i.e., control). Another subject with a 10th-grade education scored below control (20/30). A severe TBI patient (C) scored low (9/30), although both Figures 2B,C have 10th-grade education, are female and are close in age (26 and 21 respectively). For all subjects, this test clearly stratified performances better than others, as shown in the other figures, such as Figures 6A, 7A.

### Bell’s test

In Bell’s test, subjects must find and circle all 35 bell-shaped images (circled red in Figure 3A). The test seems very easy for most subjects despite their conditions, except for the minor indication that controls and mild TBI patients have more or less compact response ranges (oscillating around the maximum score of 35, see Figure 6C). Nevertheless, there were a few clear examples, such as a moderate TBI patient who missed many bells around the left bottom half of the image maze (in Figure 3B, red dots indicate missed ones), suggesting spatial neglect of the left bottom quadrant of the visual field.

### Line-bisection test

The line-bisection test performs well (second best paper test, first being MoCA), especially in finding visual deficits. Figures 4B,C exhibit severe cases of left-sided neglect, Figure 4D a right-sided neglect, while Figure 4A, a control subject, shows only a few minor errors. Figures 4C,D also failed to cross the lines numbered 4 and 1, respectively (red dots on the right side of the paper denote missed lines).

### Education level and paper tests

**Figure 8:**
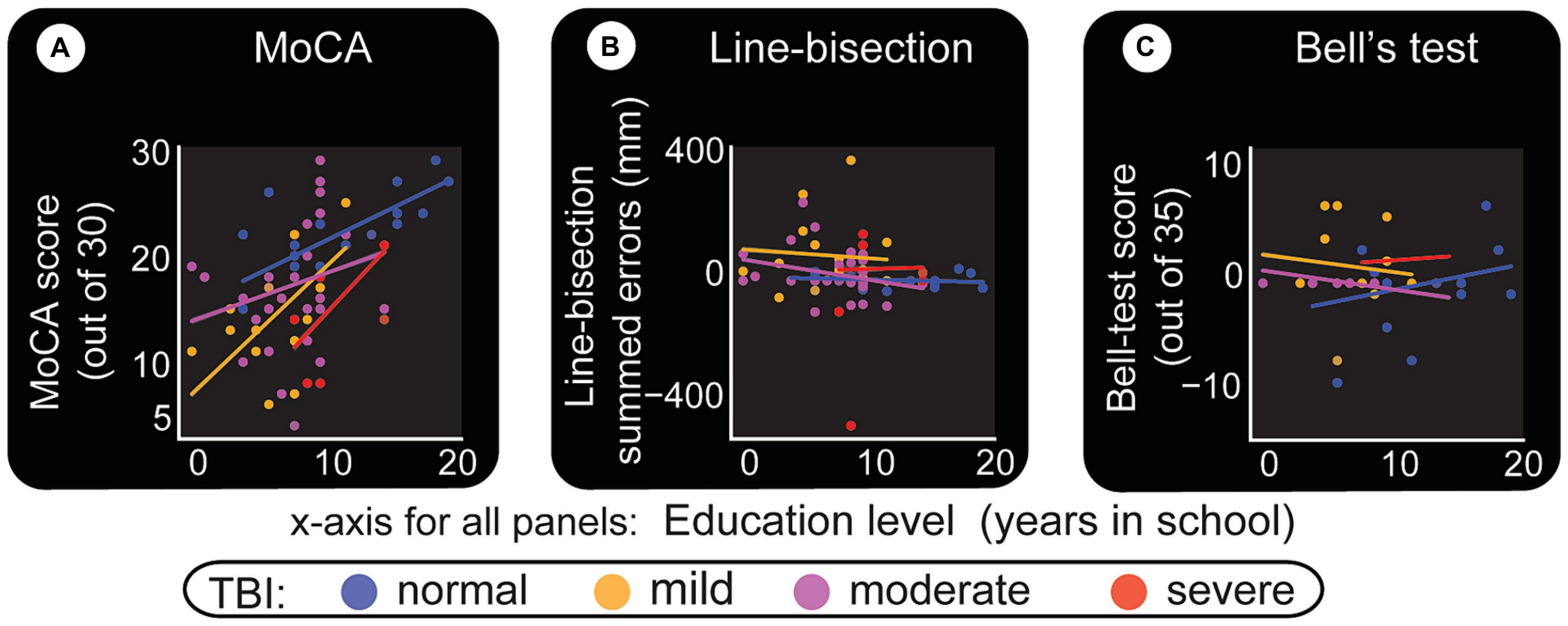
Level of education and its relationship with paper test performance. Level of education as described by years in school is the x-axis for all panels. **(A)** MoCA test. Despite the limited number of participants, this test shows an education effect, but similar for all classes of TBI. **(B)** Line-bisection test. Y-axis quantifies the sum of all errors (in millimeters) that are to the left (negative) and right (positive) of the ideal middle point. More positive numbers indicate left neglect. **(C)** Bell’s test. A score of 35 indicates that the subject has found and circled all bell-shaped images. This result indicates that most can perform the test relatively well. It is interesting to note however that, for both line-bisection and Bell’s tests, normal subjects appear to have similar (stable) responses at all education levels. Also, there seem to be minimal line-bisection errors [errors are around 0 to +/− 50 pixels in **(B)**, also see Figure 6B]. Note the one outlier severe TBI patient with right neglect (actual test result shown in Figure 4D). Bell’s test results for normal subjects also appear to have a straight fit line that hovers approximately above 30 (also see Figure 6C).

Results in this area were classified by education level and TBI to assess bias in the paper test. Figure 8A confirms that MoCA displays an educational effect, and it behaves the same way for all classes of TBI. i.e. higher education level corresponds to better performance. Line-bisection (Figures 8B, 6B) and Bell’s test (Figures 8C, 6C) both show that control subjects have a stable error distribution and test performance, respectively with limited education effect. For line-bisection, control subject errors stay around 0 and are less chaotic in their distribution (in comparison, the subject in Figure 4D, showed huge negative errors).

A deeper look at the relationship between TBI categories, paper tests, and education level is depicted by boxplots in Figure 6. As a general indication of the trends the data is going toward, the figure shows statistical results from multiple comparisons between the categories performed using Mann–Whitney tests with Bonferroni corrections. This approach is used when a nonparametric test is required to compare two independent groups (i.e., to test whether two samples are likely to derive from the same population). A nonparametric test is chosen also because of the small sample size, which makes it difficult to be certain about the kind of distribution the general population has. The boxplots in Figure 6 show that the medians (which this test compares) vary between groups. The level of certainty (differences) is depicted by asterisks (‘*’) or ‘ns’ (no significant differences) as shown in the cartoon in the bottom right.

Almost all paired comparisons show significant differences, especially when TBI groups are compared to controls.

MoCA results (Figure 6A) indicate that TBI severity decreases test performance. Control subjects have higher scores, near symmetric whiskers, and a slight positive skewness.

Figure 6B summarizes the line-bisection test scores versus TBI level. The boxplots are plotted against a *Y*-axis which depicts the difference between counts of right (positive) crossings of the ideal middle for each line minus negatively skewed crossings. In other words, more negatively skewed crossings indicate possible right-sided neglect. Here, as in the other tests, it is imperative to not overinterpret the statistical significances as concrete proofs but rather as early indications of trends that will need to be proven at a later time when larger data sets are available.

Bell’s test score versus TBI does not show a major effect (Figures 8C, 6C). Education level does not appear to be related to TBI damage, except that controls tend to have higher education (Figure 6D), possibly due to one or more of several reasons. As shown in Figure 6E, alcohol consumption is not similar between the different groups (for instance controls tend to be the least consumers), although the present sample size does not warrant profound conclusions.

### Computer test results

Almost all subjects were also tested on computer games measuring vision, memory, math, and other functions. The results were compared to paper tests and other metrics to understand similarities, differences, and interactions. Figure 7 summarizes these findings by using categorical line plots. Each computer test result is plotted against a paper test or another variable to show the central tendency of the data points (their mean) and the errors around them. They show how each outcome changes based on the different TBI categories. For instance, we can investigate how Stroop test results change with the TBI category, how they compare with MoCA results and what their relationship with the same categories is. Imagine each line as a group data (mean) for subjects who took that computer test.

The first and most significant finding is that the tests are performed dissimilarly, and test accuracy decreases with TBI severity. Figure 7E depicts test accuracy (correctness). Y-axis indicates the average accuracy (scaled 0 to 1) for the given cohort. The score of “1” indicates all group members (for example, control subjects doing RGB task) scored perfectly. We observe that for each cohort and test type, the standard error around the mean performance increases with TBI severity (indicating variability in individual deficits).

Almost all computer test results show a declining MoCA score as TBI severity increases. This relationship between a computer test and TBI category is one of our most precise results and is shown in Figure 7A. Individual performance on MoCA tracks performance on all computer tests.

Figure 7B plots computer test results against line-bisection errors. Two observations are worth noting here. (1) Control subjects have similar results for each test and less error around the mean values. (2) TBI groups show inter-test variability and larger errors around the mean for each test. The same two general observations hold for Bell’s test (Figure 7C) with one caveat. With Bell’s test, control subjects perform differentially on each computer test. Whether this is due to their better visual performance (which this test examines) or any other reason remains to be seen.

### Visually and memory-guided hand movement errors

Next, we look at vision and memory-guided movements, two brain functions whose disruptions often cause serious life complications in TBI patients. The results indicate that visually-guided hand movements are more accurate.

**Figure 9:**
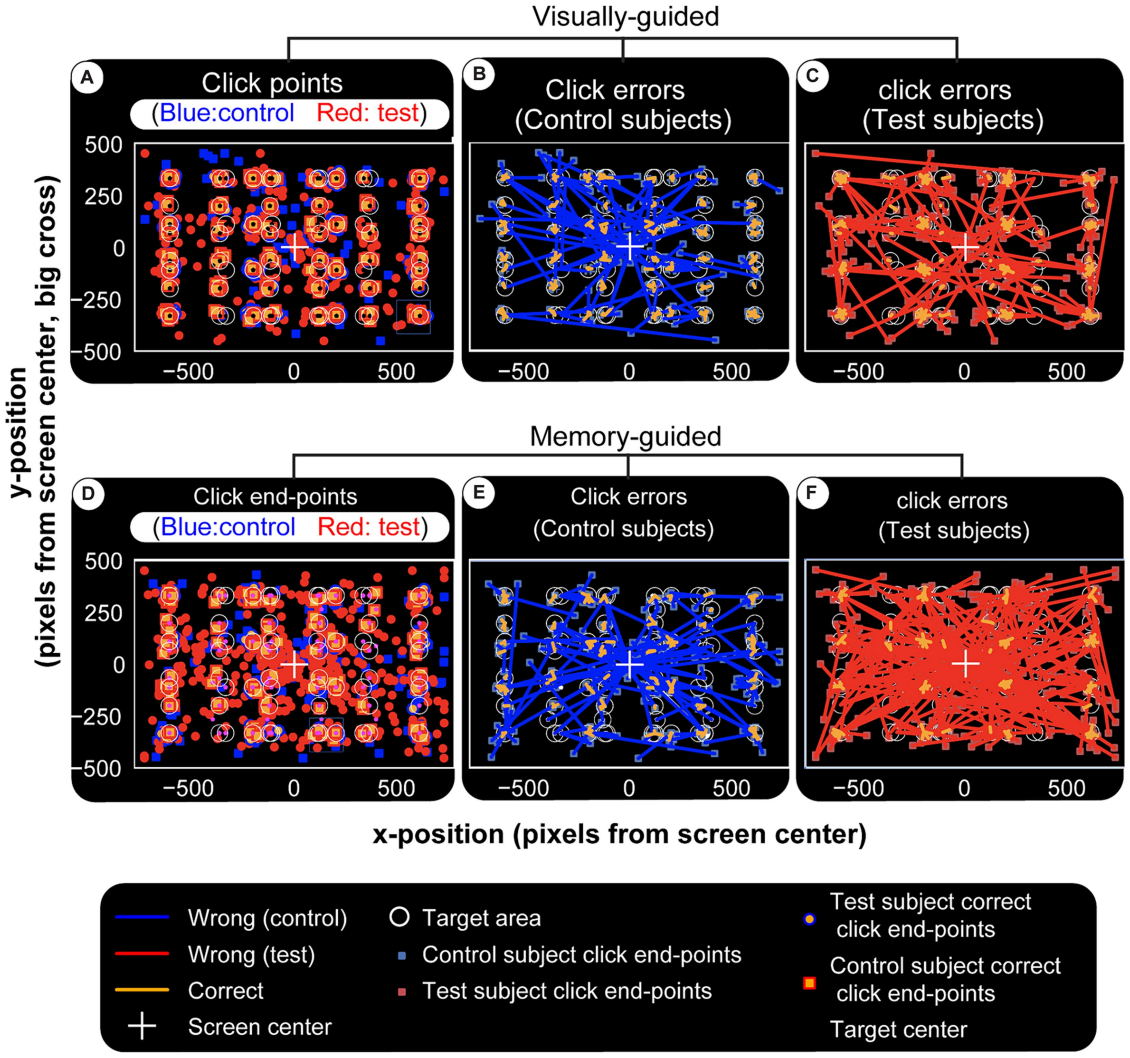
Visual and memory-guided click errors. Subjects are required to hold the computer cursur at the center of the screen (big white cross) and move to a 40 pixel radius circular visual target (white circles) when it appears in a random location. They reach to it with the cursor while it is still on (visual) or after it disappears (memory). Once at the target location, the subject clicks the mouse, trial ends. Blue squares and red circles indicate wrong click points (outside the targer area) for control and test subjects, respectively. After a random inter-trial interval, the central fixation cross appears again for th next trial, with the computer cursor automatically reset to the same location. Panel **(A–C)** are for visual and **(D–F)** are for memory-guided experiments. **(A,D)** Show correct and wrong click points. Blue and red lines in **(B,C,E,F)** are drawn from the actual click points to where they were supposed to be made. They depict the maginitude of the errors (distance from center of target circle) for visual and memory targets in controls and test subjects, respectively. While both groups make larger errors for memorized targets, test subjects (patients) have higher error rates and magnitudes, again especially for memory targets. Visually **(F)** is more crowded than **(E)** and this error magnitude is quantified in Figure 10A.

**Figure 10:**
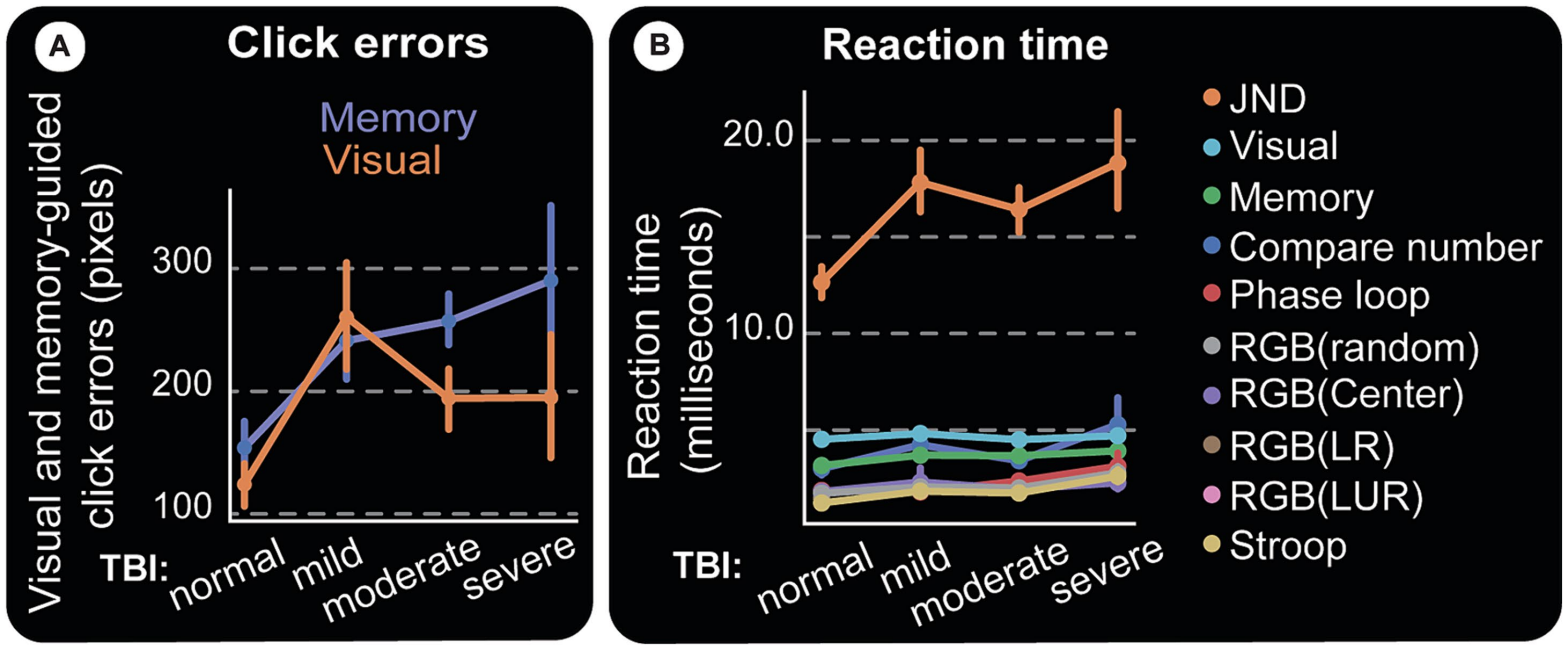
Clicking errors and reaction time. **(A)** Magnitudes of vision and memory-guided click errors away from the center of the target circle. Moderate and severe TBI subjects have almost twice as much errors as control subjects, especially for memory-guided clicks. **(B)** Reaction Time. Response time (RT) starts at stimulus presentation, includes reaction and movement time, until the completion of the required response. For instance, deciding which one of two almost similar images that appear flanking a central fixation point is not slanted (our JND experiment), is a difficult task and should take a long time. Indeed, the figure shows it to be the longest response time computer test amongst all we administered and at least with the limited data we have, does not track TBI.

The subject is required to move the cursor to the center of the target circle and click. Thus, error is measured as the distance between this ideal coordinate and the response (end point). Error distributions for visual and memory tasks show two primary effects: (1) controls make fewer errors; (2) patients’ errors are larger (Figure 9). End-points (blue for controls and red for patients) in panels Figures 9A,D are numerous and far from the invisible target circles (white) for the TBI victims. These differences are shown by error lines drawn between the target and response. It is visually clear that controls have a more or less similar error profile (Figures 9B,E), while the patient group makes longer errors even in visual experiments (Figure 9C) and an effect more pronounced in the memory version (visually compare Figure 9C and Figure 9F). This is summarized in Figure 10A, which displays the mean values for each TBI group and experiment. It shows an increasing trend based on injury severity and that memory errors are about twice that of visually-guided responses (for all TBI groups).

### Computer test response times

Computers allow us to measure subjects’ response speed, which can be affected by factors such as task difficulty, visual and/or motor deficits, age, etc. The computer tests show an overall increase in response time (RT) as TBI severity goes up (Figure 10B).

## Discussion

Given the lack of trained professionals and modern diagnostic tests/devices, such as diffuse tensor imaging, x-rays, and CT scans, the projected increasing burden from TBIs and subsequent disabilities within LMICs, and the likelihood that such deficits will in turn lead to work-related accidents or injuries, developing simple, accurate, and scientifically based tests that help identify post-TBI deficits is essential. These tests should be simple, affordable, and give the clinician meaningful and accurate evidence of measurable neurological TBI related deficits. This information can assist clinicians to isolate TBI patients with either memory or visual deficits, or both who may benefit from close follow-up and rehabilitation before returning to work.

Our results from both paper and computer tests show a similar trend - that TBI severity reduces performance in all tests.

It is important to make clear that the tests and results are not to be taken as adequate and conclusive. There are few points to consider in pushing the research forward. First, the situation on the ground in Ethiopia did not allow to repeatedly bring patients back to the hospitals and perform a longitudinal study as most subjects traveled hours and some even days to be tested.

Second, patients that could be included were in various, post-TBI stages days to months and had a mix of mild, moderate, and severe TBI levels, making it hard to come up with a sample size to cover the full time frame and the three levels on TBI injury. These two challenges are demonstrated in the figure in Supplementary Appendix B, which shows at the time of test administration injury from 1 day to 115 days on average is included in the patient population. The length of injury contained most (38/51) below the 75th percentile for the cohort (240 days since injury), and 50th percentile is 115 days. All TBI groups have a more-or-less similar distribution.

These two main obstacles made it impossible to perform the tests repeatedly on a single subject and also to include other examinations like those which probe post-traumatic amnesia (PTA). However, the implemented tests are good starting points and mostly deal with the important questions, for instance, MoCA indirectly covers most of the PTA questions.

In keeping with its reputation, the MoCA test resulted in a clear separation between control and various TBI groups. All analysis points toward a possible use in the clinic for testing a few important brain functions such as comprehension, memory, and simple math.

Some tests, such as Bell’s test, show inferior performance, perhaps because there were only 71 subjects in this study and also due to TBI related reasons. For instance, Figures 4B–D (the line-bisection test) and a few other patients, although they are otherwise visually normal and can make a few correct cancelations, seem to make impulsive decisions and hastily cross the lines in what appears to be a straightforward pattern, with similar horizontal location for all lines.

Despite the experiments being designed to test different mental and visual capacities, TBI patients may follow the same strategy to answer test questions. These could include impulsive and ballistic cursor movements to a visual target to maximize the chances of hitting it without regard to accuracy of where in the large circle the end point is, use the pencil to do a very slow horizontal scanning on Bell’s test, keep saying ‘higher’ for a consecutive numbers comparison test, etc.

One trend we noticed is that mild and moderate TBI patients line-bisection errors seem to reduce with more education (downward slopping green and blue lines in Figure 8B). The word ‘trend’ is used here to indicate that more subjects need to be included (per our calculations, at least 200 subjects) for statistical power. Multiple comparison tests (Figure 6A) indicate that there are differences that need to be verified further. Once again, tests like MoCA (Figure 9A) and memory-guided hand (computer cursor) movements (Figures 9D,E) indicate deficits caused by TBI.

Depending on which parts of the brain are affected by the specific TBI, visually-guided saccades [which involve early visual areas (55)] could be affected separately from memory-guided saccades, which are slower by about 100 ms and involve more cortical processing and other brain regions (56). The same applies to hand movements. The effects can be even more complicated if the TBI affects an area of the cortex involved in both arm and hand movements. In our results, however, visually guided movements are slower than memory-guided movements (Figure 10B).We attribute this difference in visual vs. memory guided movements to task design for two reasons. First, because subjects were able to plan movements during the memory period. We were only concerned about accuracy. Second, the subjects, especially moderate or severe TBI patients, were impatient and too eager to respond, especially on memory trials.

Computer tests tracked TBI deficits and MoCA performances, suggesting possible objective test development and even future rehabilitation exercise creation.

Most of our patients had injuries from violence than from RTA or alcohol abuse, maleness is a precipitating factor consistent with the data from most of the world (54), in addition to young age and violence. In Ethiopia, a meta-analysis showed that across all studies there is higher proportion of injury in male than female patients (range 53.9–91.2%) and in economically active age groups of 15–59 years (Range 56.4–80%) (34).

These facts call for concerted efforts to change the circumstances and allow improved measures to quantify the outcomes. There is high enthusiasm in the patient population, practicing medical professionals, and high-ranking officials for further research as the tools that can develop would help to objectively assess injury-related deficits that are mostly missed by the regularly used physical tests. If proven useful, this comprehensive tool can both make insurance claim and discharge policies better suited for all involved as well as improve the quality of life of patients with TBI. For example, drivers who sustain a TBI, could be required to take comprehensive tests such as those presented here, before being allowed to return to work.

Data acquisition will continue to include a large cohort for statistical analysis and to research the three TBI classes and the related specific deficits and appropriate tests. Of these, mild TBI needs particular focus as it tends to be ignored or misdiagnosed, covers 90% of TBI cases presenting to hospital (6), and its symptoms are not usually regarded as serious, even when patients and their families raise credible concerns.

The project will be expanded to include more subjects, to collect a statistically validated large dataset, and to generalize the results to different populations, hospital settings, and LMICs. Other tasks, such as mini-Mental State Exam (MMSE), true/false sentences, and attention studies using disengage tasks such as anti-saccades, etc., will also be included.

## Study limitations

The study presents promising evidence that the employed diagnostic tests could serve as practical assessment tools for Traumatic Brain Injury (TBI) in Low- and Middle-Income Countries (LMICs). Nonetheless, the study confronts several inherent limitations that temper the conclusiveness of its findings. These include a limited participant pool, gender imbalances, and an uneven representation of TBI severity levels.

Furthermore, the study’s environment faced logistical challenges, with many hospitals experiencing shortages of basic facilities such as rooms, desks, and even chairs—often leading to medical staff overseeing the procedures while standing. In designing the tests for such constrained settings, there was minimal regulation of external variables like hospital staff interruptions or ambient noise.

Acknowledging the likelihood of participants’ unfamiliarity with paper-based and computerized tests, we took extensive measures to provide clear instructions. Testing commenced only after ensuring participants’ comprehension and ability to engage fully with the test materials.

In terms of assessment tools, although the Glasgow Coma Scale (GCS) is a recognized predictor of TBI outcomes, it, along with the Glasgow Outcome Scale (GOS) and the extended version (GOS-E), was not used in this study to gauge progress for several reasons. Primarily, the operational context in Ethiopia does not support regular patient follow-ups or standardized treatments for TBI outside of necessary surgical interventions. This means that treatment outcomes depend heavily on individual circumstances. Additionally, the use of GOS and GOS-E is not widespread due to the absence of national treatment standards and a shortage of professionals trained to administer these examinations.

Further research with inclusion of a large cohort and additional tools is necessary to work around these limitations and establish tools and best practices.

## Conclusion

Our main findings demonstrate that a simple paper and computer test, implemented by one experimenter can provide useful results to the clinician in assessing several TBI-injury-related deficits in resource-limited settings such as in LMICs. Other findings include: subject demographic data such as education level, which can have significant effects on test performance; alcohol consumption and the possibility of being exposed to TBI incidences. These facts call for collaborative efforts to change the circumstances and allow improved measures to combat the problems. More data with a large sample size is required to appreciate the accuracy of the combined testes in identifying post-TBI deficits. The decision to classify a patient as belonging to one of the TBI categories or as ‘control’ has great implications and has to be studied carefully. Because medical tests are evaluated for specificity and sensitivity, the need for large data set is critical to ensure that these and other parameters could be estimated with great confidence.

## Data availability statement

The original contributions presented in the study are included in the article/Supplementary material, further inquiries can be directed to the corresponding author/s.

## Ethics Statement

The studies involving humans were approved by Black Lion Specialized Hospital, Department of Neurosurgery, Addis Ababa, Ethiopia Neurosurgery Unit, College of Health Science. The studies were conducted in accordance with the local legislation and institutional requirements. Written informed consent for participation in this study was provided by the participants’ legal guardians/next of kin.

## Author contributions

MS: Writing – review & editing, Writing – original draft, Visualization, Validation, Supervision, Software, Resources, Project administration, Methodology, Investigation, Funding acquisition, Formal analysis, Data curation, Conceptualization. TL: Writing – review & editing, Supervision, Resources, Project administration, Investigation, Funding acquisition. AA: Writing – review & editing, Supervision, Resources, Project administration, Investigation, Funding acquisition, Conceptualization. AT: Writing – review & editing, Supervision, Resources, Project administration, Investigation. YA: Writing – review & editing, Supervision, Resources, Project administration, Investigation. PT: Writing – review & editing, Resources, Project administration, Supervision, Investigation. AK: Writing – review & editing, Resources, Project administration, Methodology, Funding acquisition, Conceptualization. PH: Writing – review & editing, Resources, Project administration, Methodology, Funding acquisition, Conceptualization. AB: Writing – review & editing, Supervision, Resources, Project administration, Investigation. DY: Writing – review & editing, Supervision, Resources, Project administration, Investigation. GH: Data curation, Conceptualization, Writing – review & editing, Validation, Supervision, Resources, Project administration, Methodology, Investigation, Funding acquisition.
