# Supplementary material for: Extended tests for evaluating post-traumatic brain injury deficits in resource-limited settings: methods and pilot study data
Source: Front Neurol. 2024 Jun 12;15:1397625. doi: 10.3389/fneur.2024.1397625 (PMC11199529; doi:10.3389/fneur.2024.1397625)
Supplement: Supplementary file 1 [file Table_1.DOCX]

| Group | Age | Gender | Education level | TBI | cause | Affected side | MoCA | Line bisection | Bell's |
| --- | --- | --- | --- | --- | --- | --- | --- | --- | --- |
| control | 32 | male | 12 |  |  |  | 22 | -34 | 35 |
| control | 26 | female | 10 |  |  |  | 20 | -29 | 34 |
| control | 26 | female | 20 |  |  |  | 28 | -24 | 33 |
| control | 30 | male | 10 |  |  |  | 27 | -23 | 35 |
| control | 33 | male | 16 |  |  |  | 24 | -22 | 14 |
| control | 44 | female | 16 |  |  |  | 25 | -11 | 35 |
| control | 31 | male | 12 |  |  |  | 23 | 0 |  |
| control | 26 | male | 4 |  |  |  | 16 | 0 | 35 |
| control | 36 | male | 10 |  |  |  | 19 | 0 | 33 |
| control | 39 | male | 16 |  |  |  | 28 | 0 | 35 |
| control | 62 | male | 8 |  |  |  | 20 | 0 | 0 |
| control | 35 | female | 10 |  |  |  | 19 | 4 | 35 |
| control | 28 | male | 10 |  |  |  | 24 | 8 | 35 |
| control | 43 | male | 9 |  |  |  | 21 | 14 | 34 |
| control | 20 | male | 6 |  |  |  | 27 | 24 | 35 |
| control | 25 | female | 19 |  |  |  | 30 | 25 | 33 |
| control | 26 | female | 8 |  |  |  | 21 | 28 | 31 |
| control | 65 | female | 6 |  |  |  | 18 | 30 | 16 |
| control | 24 | female | 18 |  |  |  | 25 | 39 | 33 |
| control | 25 | male | 8 |  |  |  | 22 | 53 | 34 |
| test | 40 | male | 3 | mild | violence | right | 16 | -55 | 34 |
| test | 29 | male | 14 | mild | RTA | right | 18 | -32 | 26 |
| test | 28 | male | 9 | mild | fall | left | 15 | 0 | 34 |
| test | 43 | male | 12 | mild | RTA | left | 26 | 0 | 34 |
| test | 25 | male | 10 | mild | RTA | both | 18 | 8 | 35 |
| test | 39 | male | 8 | mild | violence | right | 13 | 27 | 31 |
| test | 45 | female | 0 | mild | RTA | left | 12 | 31 | 25 |
| test | 22 | male | 10 | mild | violence | right | 19 | 35 | 33 |
| test | 30 | male | 8 | mild | RTA | right | 19 | 36 | 30 |
| test | 35 | male | 8 | mild | violence | right | 23 | 52 | 25 |
| test | 24 | male | 3 | mild | violence | left | 14 | 57 | 28 |
| test | 30 | male | 8 | mild | RTA | both | 8 | 71 | 32 |
| test | 25 | male | 6 | mild | violence | right | 7 | 117 | 31 |
| test | 45 | male | 12 | mild | violence | left | 23 | 125 | 35 |
| test | 23 | male | 5 | mild | violence | right | 14 | 162 | 35 |
| test | 40 | male | 5 | mild | violence | right | 12 | 282 | 29 |
| test | 47 | male | 9 | mild | RTA | right | 16 | 393 | 33 |
| test | 50 | male | 6 | moderate | violence | left | 19 | -102 | 29 |
| test | 36 | male | 12 | moderate | violence | both | 23 | -83 | 32 |
| test | 27 | male | 9 | moderate | RTA | left | 16 | -80 | 30 |
| test | 24 | male | 10 | moderate | violence | left | 30 | -77 | 35 |
| test | 26 | male | 10 | moderate | fall | right | 17 | -20 | 31 |
| test | 40 | female |  | moderate | RTA | both | 16 | -10 | 28 |
| test | 21 | male | 10 | moderate | violence | right | 27 | -7 | 35 |
| test | 28 | male | 10 | moderate | RTA | both | 17 | -6 | 33 |
| test | 14 | female | 9 | moderate | violence | right | 21 | -6 | 34 |
| test | 35 | male | 8 | moderate | fall | left | 5 | 0 | 0 |
| test | 35 | male | 6 | moderate | RTA | left | 16 | 0 | 33 |
| test | 23 | female | 0 | moderate | RTA | left |  | 0 | 33 |
| test | 22 | female | 7 | moderate | RTA | left | 8 | 0 | 23 |
| test | 16 | male | 4 | moderate | violence | left | 17 | 0 | 34 |
| test | 39 | male | 9 | moderate | violence | right | 13 | 0 | 35 |
| test | 28 | male | 10 | moderate | violence | right | 27 | 0 | 33 |
| test | 16 | male | 7 | moderate | RTA | left | 17 | 1 | 35 |
| test | 24 | male | 1 | moderate | violence | right | 19 | 14 |  |
| test | 38 | male | 10 | moderate | RTA | right | 11 | 29 | 23 |
| test | 20 | male | 8 | moderate | fall | right | 19 | 53 | 27 |
| test | 15 | male | 9 | moderate | violence | right | 24 | 59 | 32 |
| test | 28 | male | 10 | moderate | fall | left | 25 | 67 | 35 |
| test | 32 | male | 10 | moderate | violence | both | 28 | 86 | 34 |
| test | 24 | male | 0 | moderate | violence | right | 20 | 88 | 33 |
| test | 23 | male | 9 | moderate | violence | left | 21 | 93 | 31 |
| test | 25 | male | 4 | moderate | violence | left | 11 | 133 | 8 |
| test | 30 | male | 6 | moderate | RTA | left | 12 | 175 | 31 |
| test | 35 | male | 5 | moderate | fall | both | 15 | 255 | 34 |
| test | 35 | male | 0 | severe | violence | left | 9 | -473 | 33 |
| test | 29 | male | 8 | severe | violence | right | 15 | -101 | 28 |
| test | 28 | male | 15 | severe | fall | left | 22 | 0 | 35 |
| test | 45 | male | 15 | severe | RTA | left | 15 | 26 | 32 |
| test | 45 | female | 10 | severe | RTA | right | 19 | 116 | 34 |
| test | 21 | female | 10 | severe | RTA | left | 9 | 152 | 32 |

**Appendix A.** Subject demographics and paper test results.
