# Supplementary figures and images for: Extended tests for evaluating post-traumatic brain injury deficits in resource-limited settings: methods and pilot study data
Source: Front Neurol. 2024 Jun 12;15:1397625. doi: 10.3389/fneur.2024.1397625 (PMC11199529; doi:10.3389/fneur.2024.1397625)

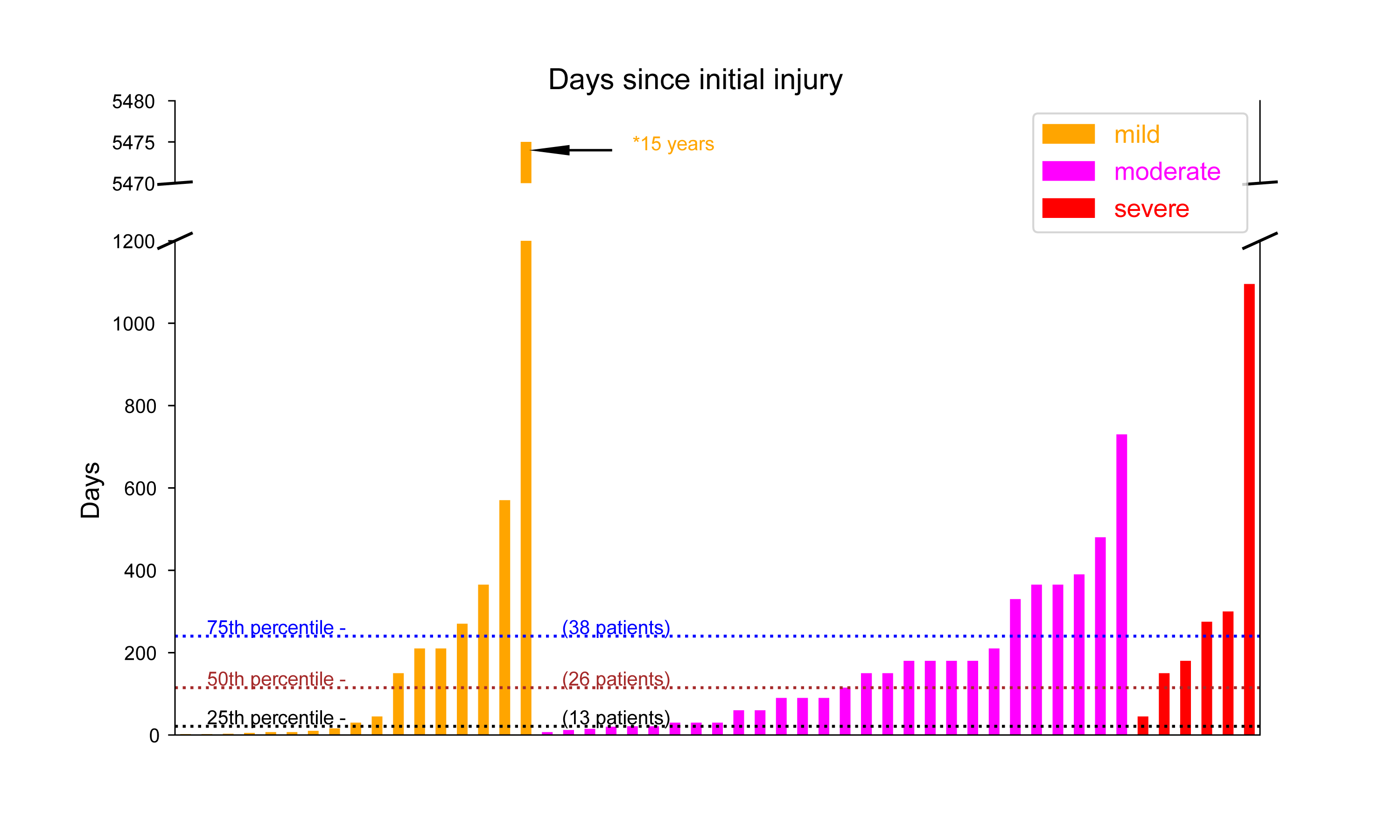

Supplement: Supplementary file 2 [file Image_1.TIF]
